# Pentraxin-3 as a marker of disease severity and risk of death in patients with necrotizing soft tissue infections: a nationwide, prospective, observational study

**DOI:** 10.1186/s13054-016-1210-z

**Published:** 2016-02-15

**Authors:** Marco Bo Hansen, Lars Simon Rasmussen, Peter Garred, Daniel Bidstrup, Martin Bruun Madsen, Ole Hyldegaard

**Affiliations:** Department of Anesthesia, Center of Head and Orthopedics, Rigshospitalet, University of Copenhagen, Blegdamsvej 9, Copenhagen, DK-2100 Denmark; Hyperbaric Unit, Department of Anesthesia, Center of Head and Orthopedics, Rigshospitalet, University of Copenhagen, Blegdamsvej 9, Copenhagen, DK-2100 Denmark; Laboratory of Molecular Medicine, Department of Clinical Immunology, Rigshospitalet, University of Copenhagen, Blegdamsvej 9, Copenhagen, DK-2100 Denmark; Department of Intensive Care, Rigshospitalet, University of Copenhagen, Blegdamsvej 9, Copenhagen, DK-2100 Denmark

**Keywords:** Sepsis, Amputation, Survival, Procalcitonin, C-reactive protein, Necrotizing fasciitis

## Abstract

**Background:**

New biomarkers are needed to assess the severity of necrotizing soft tissue infection (NSTI) at an early stage and to individualize treatment strategies. We assessed pentraxin-3 (PTX3) as a marker of disease severity and risk of death in patients with NSTI.

**Methods:**

We conducted a prospective, observational study in the intensive care unit at Copenhagen University Hospital, where treatment of NSTI is centralized at a national level. We compared PTX3, procalcitonin and C-reactive protein in septic shock versus nonshock patients and in amputated versus nonamputated patients using the Mann-Whitney *U* test. The prognostic value of the markers for 180-day mortality was assessed using Cox regression analyses.

**Results:**

Patients with NSTI (n = 135) were included over 25 months with up to 2.5-year follow-up; 71 % had septic shock, amputation was undertaken in 20 % and the 180-day mortality was 27 %. Baseline plasma PTX3 level was significantly higher in patients with septic shock (67.3 versus 24.6 ng/mL, *p* < 0.0001) and in patients who underwent amputation (118.6 versus 43.6 ng/mL, *p* = 0.019). No significant differences in baseline procalcitonin or C-reactive protein levels were found according to amputation (25.2 versus 7.0 μg/L, *p* = 0.060 and 202 versus 225 mg/L, *p* = 0.123), respectively. Baseline PTX3 level above the median was associated with death (*p* = 0.009, log-rank test) and the univariate Cox regression analysis revealed a significant association between PTX3 level upon admission and 180-day mortality (hazard ratio 2.60 (95 % confidence interval 1.28–5.29), *p* = 0.008). When adjusted for age, sex, chronic disease and Simplified Acute Physiology Score II, no significant association was found.

**Conclusions:**

High PTX3 level is associated with septic shock, amputation and risk of death in patients with NSTI, but it is not an independent predictor of 180-day mortality in this patient group.

**Trial registration:**

ClinicalTrials.gov Identifier: NCT02180906. Date of registration: June 29, 2014.

## Background

Necrotizing soft tissue infection (NSTI) is a bacterial infection of any layer within the soft tissue compartments associated with necrosis. The condition is often accompanied by septic shock, and the extensive inflammatory response is thought to be a primary cause of mortality [1]. Delay of surgery has been shown to be an independent risk factor for mortality, and studies stress the importance of surgical debridement and early amputation of infected limbs [2–5]. However, an aggressive surgical approach increases the risk of severe disability and impaired quality of life. Moreover, surgical decisions are often based on the surgeon’s experience and practice guidelines.

Biomarkers may provide therapeutic guidance and prognostication, and thereby improve decision-making in patients with NSTI. To date, C-reactive protein (CRP) and procalcitonin (PCT) have been used to monitor infectious disease progression in the intensive care unit (ICU). However, the literature investigating the value of biomarkers in patients with NSTI is sparse and often limited by retrospective study designs [6–8]. Due to the low incidence, the knowledge and evidence from septic patients are extrapolated to those with NSTI, even though the immunological reaction may differ because of the extensive tissue damage and different pathogenesis. It would be desirable if biomarkers could be used to identify patients with NSTI at high risk of death, as more aggressive treatment could then be undertaken in this subgroup while extensive surgery could be avoided in low-risk patients.

Pentraxin-3 (PTX3) is a multifunctional pattern-recognition molecule released at the onset of inflammation as part of the innate immune system by activating the classical and lectin complement pathways through specific recognition of the C1q, mannose-binding lectin, ficolin-1 and ficolin-2 subunits [9–13]. A high PTX3 level is associated with disease severity and mortality in patients with myocardial infarction [14–16], ischemic stroke [17], cancer [18, 19], acute respiratory distress syndrome [20] and sepsis [21–23]. PTX3 is closely related to CRP as both are members of the pentraxin family of proteins, and PTX3 is central to the antimicrobial responses and in the clearance of cellular debris.

As PTX3 is produced locally by various cells, including monocytes, neutrophils and endothelial cells in response to tumor necrosis factor and bacterial products, it is reasonable to consider PTX3 as a marker of local inflammation detected by direct release to the blood stream [24]. In contrast, CRP is produced in the liver in response to locally induced interleukin-6 [25, 26], while the expression and synthesis of PCT in response to inflammatory stimuli seems to be more ubiquitous [27]. Despite the promising role of PTX3 in the risk stratification of various infections and inflammatory conditions, the prognostic relevance in patients with NSTI remains to be evaluated.

The aim of this study was to assess plasma PTX3 as a marker of disease severity and risk of death in patients with NSTI.

## Methods

### Study design and setting

This prospective, observational study was conducted during February 2013 to March 2015 at Copenhagen University Hospital (Rigshospitalet) as a substudy of the ongoing European INFECT project (ClinicalTrials.gov Identifier: NCT01790698). The protocol of this study is described in greater detail elsewhere [28] and registered at ClinicalTrials.gov (NCT02180906). In Denmark (population 5.6 million) the treatment of NSTI is centralized at a tertiary referral hospital, Rigshospitalet, University of Copenhagen.

### Study population

#### Patients with NSTI

Patients were included if they had been: (1) diagnosed with NSTI based on surgical findings with necrosis engaging any layers of the soft tissue compartments; (2) aged ≥18 years; and (3) had either been admitted to the ICU or undergone surgery for NSTI at Rigshospitalet. Patients were excluded if the NSTI diagnosis could not be confirmed during surgery. For the majority of patients, the first operation had been at a primary hospital before the transfer to our center.

#### Control patients

Control patients were eligible for inclusion if they were: (1) undergoing elective orthopedic surgery at Rigshospitalet; and (2) were aged ≥18 years. Patients with ongoing infection or inflammatory conditions were excluded. We included 65 control patients, matched for age and sex during September 2014 to March 2015.

### Data collection

#### Clinical data

We obtained data from electronic records on age, sex, body mass index, chronic disease (diabetes, liver cirrhosis, chronic kidney disease, cardiovascular disease, chronic obstructive pulmonary disease, peripheral vascular disease, immune deficiency, malignancy, rheumatoid disease), primary site of infection, microorganism, biochemistry, ICU scoring systems and treatment. Vital status and time of death, if relevant, were extracted from the hospital database linked to the Danish Civil Registration System. The criterion for amputation was based on the surgeon’s clinical evaluation of the infected body part and performed as a final method to obtain infection control. No strict protocol for amputation was used.

#### Blood sampling and processing

Blood was drawn from an arterial line into 9-mL vacuum tubes containing EDTA at four discrete time points: on admission (baseline) and the following three days between 8 a.m. and 12 a.m. For the control group, the blood samples were drawn by venous puncture at three discrete time points: preoperatively (baseline), 2–6 h postoperatively and the day after surgery between 8 a.m. and 12 a.m. The blood sample was immediately put on ice until plasma was separated from the whole blood by centrifugation (within 40 minutes) at 3500 rpm (2400 G) for 10 minutes and was subsequently stored at −80 °C.

#### Routine biochemistry and hematology

Standard blood analyses including platelet count, creatinine, leucocyte count, PCT and CRP levels were performed at the Department of Clinical Biochemistry, Rigshospitalet, as part of routine analyses, whereas sodium, potassium, hemoglobin, lactate, pH, base excess, pO_2_ and pCO_2_ were measured using an ABL 725 (Radiometer, Copenhagen, Denmark).

#### Enzyme-linked immunosorbent assay

Plasma PTX3 levels were determined using anti-PTX3 monoclonal antibodies in a sandwich enzyme-linked immunosorbent assay (ELISA) developed in the Laboratory of Molecular Medicine, Rigshospitalet, according to previously described procedures [23, 29]. In short, microtiter plates (Nunc Immuno Plates, F384 Maxisorp) were coated with 2 μg/mL mouse monoclonal anti-PTX3 antibody (clone PTX-66) produced inhouse [23], diluted in phosphate-buffered saline + 0.05 % Tween-20 (PBS-T), and incubated overnight. The plasma samples were diluted with sample buffer (1:20) in triplicates and incubated for 3 h. The biotinylated anti-PTX3 detection antibody (clone PTX3-20) was diluted (1 μg/mL), applied to the wells and incubated overnight. Secondary streptavidin-horseradish peroxidase conjugate was applied for 2 h. The plates were developed with OPD substrate and hydrogen peroxide. The optical density was measured at 490 nm with an ELISA reader. Inhouse-produced biologically active recombinant PTX3 was used as a calibrator. The sensitivity of the PTX3 assay in plasma has been estimated to be 1.5 ng/mL with an intra-assay variation of 5 % and interassay variation of 10 %.

### Outcome measures

Our primary analysis focused on the association between baseline PTX3 level and disease severity by comparing plasma PTX3 level in NSTI patients with and without septic shock upon admission to our center. This comparison was performed due to an expected higher mortality rate in patients with septic shock, thereby making it a clinically relevant stratification. Septic shock was defined according to the criteria of Bone et al. [30].

Secondary analyses compared PTX3 level during the following 3 days between nonshock patients versus patients with septic shock, amputation versus no amputation, and NSTI patients versus control patients. Additionally, analyses included investigation of differences in 180-day mortality and long-term mortality up to 2.5 years between NSTI patients with high versus low levels (dichotomization by median) of PTX3, PCT and CRP. Patients were followed from the date of admission to the first of the following: death or the end of the follow-up (August 2015).

### Statistical analysis

Kolmogorov-Smirnov and Shapiro-Wilk normality tests were performed for all variables. Due to nonparametric distribution, continuous data are reported as median (interquartile range (IQR)). For categorical data we reported absolute numbers (proportions) with the use of χ^2^-test or Fisher’s exact test for comparisons. Continuous data were compared at specific time points using Mann-Whitney *U* test. Correlations were assessed using the Spearman’s rank correlation test. The prognostic value of PTX3 for long-term mortality up to 2.5 years was investigated using the log-rank test. The prognostic value of PTX3, PCT and CRP for 180-day mortality was investigated using the univariate Cox analysis. For multivariate analysis, the Cox proportional hazards regression model was used with adjustment for age, sex, Simplified Acute Physiology Score II (SAPS II) and chronic disease (yes/no). We were unable to calculate SAPS II in five patients due to missing data. These patients were excluded from the multivariate analysis. Data from the Cox analyses are presented as relative hazards with 95 % confidence intervals (CI). Area under the curve (AUC) and receiver operating characteristic (ROC) curves were reported for the inflammatory biomarkers for 180-day mortality. The optimal cutoff was identified as the value corresponding to the maximum sum of sensitivity and specificity.

*P-*values < 0.05 were considered significant. Statistical analyses were performed using Statistical Package for the Social Sciences 22.0 software (SPSS Inc., Chicago, IL, USA) and GraphPad Prism 6.0 software (GraphPad Inc., La Jolla, CA, USA).

### Sample size

On the basis of previous studies using the same analysis [23], the investigators expected a mean PTX3 level on admission of 120 ng/mL in the nonshock group and a mean PTX3 level of 210 ng/mL in the shock group. Assuming a standard deviation of 100 ng/mL, detection of this difference with a statistical power of 90 % at a 5 % significance level would require inclusion of 52 patients. The groups were expected to be unequal in size (1:4); therefore we needed to include at least 82 patients.

### Ethics

The study was approved by the regional ethics committee (H-2-2014-071) and the Danish Data Protection Agency (J. no. 30-1282) and registered at ClinicalTrials.gov (NCT02180906). Written informed consent was obtained from all patients or their legal substitute.

The manuscript was prepared according to the Strengthening the Reporting of Observational Studies in Epidemiology (STROBE) statement [31].

## Results

**Fig. 1:**
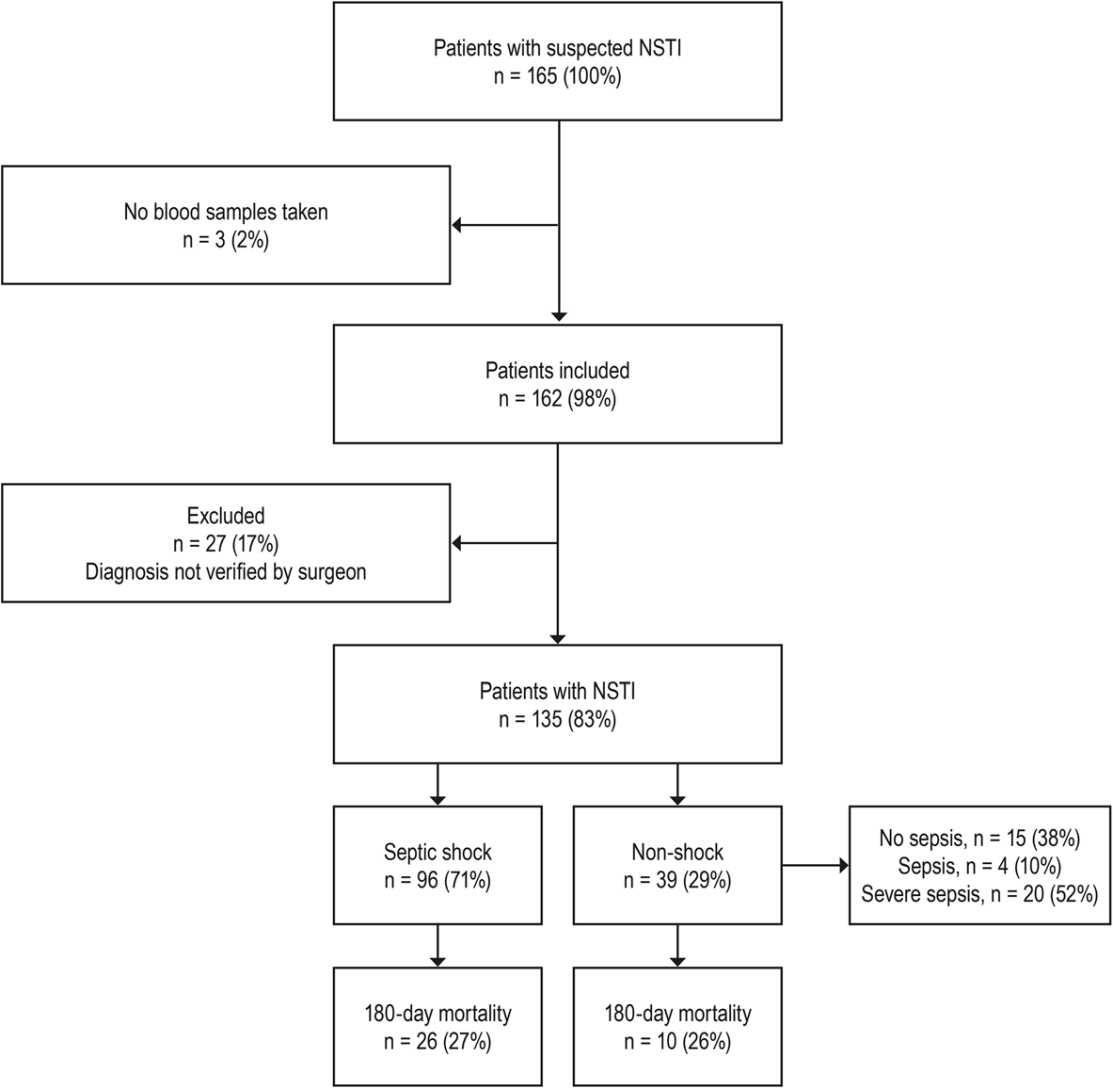
Flow chart of patient inclusion. *NSTI* Necrotizing soft tissue infection

Of 165 patients with suspected NSTI, 162 were included (Fig. 1). Subsequently, 27 patients were excluded because the NSTI diagnosis could not be verified during surgery. Thus, the study population consisted of 135 NSTI patients with a median age of 61 years (range, 52–69); 71 % (n = 96 (95 % CI, 63–78)) had septic shock, amputation was undertaken in 20 % (n = 27 (95 % CI, 14–28)) and the 180-day mortality was 27 % (n = 36 (95 % CI, 20–35)) (Tables 1 and 2).

**Table 1:** Baseline characteristics for the entire cohort of patients with necrotizing soft tissue infections and for the septic shock and nonshock (no sepsis, sepsis, severe sepsis) subgroups

	Entire cohort (n = 135)	Nonshock (n = 39)	Septic shock (n = 96)	*p*
Age, years	61 (52–69)	58 (48–70)	62 (54–69)	0.426
Sex, male	84 (62)	24 (62)	60 (63)	0.917
Body mass index, kg/m^2^	26 (23–31)	24 (22–31)	27 (25–32)	0.017
Chronic disease				
Chronic disease	86 (64)	28 (72)	58 (60)	0.213
Diabetes	30 (22)	10 (26)	20 (21)	0.543
Liver cirrhosis	5 (4)	1 (3)	4 (4)	0.655
Chronic kidney disease	12 (9)	6 (15)	6 (6)	0.104
Cardiovascular disease	58 (43)	21 (54)	37 (39)	0.103
Chronic obstructive pulmonary disease	14 (10)	3 (8)	11 (12)	0.757
Peripheral vascular disease	20 (15)	8 (21)	12 (13)	0.235
Immune deficiency/AIDS	3 (2)	2 (5)	1 (1)	0.200
Malignancy	15 (11)	3 (8)	12 (13)	0.553
Rheumatoid disease	10 (7)	2 (5)	8 (8)	0.723
Active smoker	40 (30)	9 (23)	31 (32)	0.366
High alcohol consumption^a^	19 (14)	3 (8)	16 (17)	0.191
Steroid treatment	16 (12)	4 (10)	12 (13)	0.277
Immunosuppressing drugs	12 (9)	3 (8)	9 (9)	0.281
Primary site of infection				
Head/neck	21 (15)	6 (16)	15 (16)	0.972
Upper extremity	15 (11)	3 (8)	12 (13)	0.553
Lower extremity	47 (35)	13 (33)	34 (35)	0.818
Chest	5 (4)	2 (5)	3 (3)	0.626
Abdomen	11 (8)	2 (5)	9 (9)	0.510
Genital/perineum	36 (27)	13 (33)	23 (24)	0.264
Microorganism				
Positive cultures	100 (74)	25 (64)	75 (78)	0.128
Polymicrobial	54 (40)	13 (33)	41 (43)	0.339
Beta-hemolytic streptococcus	46 (34)	9 (23)	37 (39)	0.120
Staphylococcus aureus	14 (10)	2 (5)	12 (13)	0.350
Anaerobes	23 (17)	9 (23)	14 (15)	0.312
Gram negative rods	25 (19)	6 (15)	19 (20)	0.632
Other	15 (11)	5 (13)	10 (10)	0.764

Values denote median (interquartile range) or number (%). Differences between the shock and nonshock group were tested using Mann-Whitney *U* or χ^2^-test/Fisher’s exact test

^a^High alcohol consumption: >14 units of alcohol/week (women); >21 units of alcohol/week (men)

**Table 2:** Laboratory values, clinical scoring systems and outcomes for the entire cohort of patients with necrotizing soft tissue infections and for the septic shock and nonshock (no sepsis, sepsis, severe sepsis) subgroups

	Entire cohort (n = 135)	Nonshock (n = 39)	Septic shock (n = 96)	*p*
Biomarker level upon admission (baseline)				
Pentraxin-3, ng/mL	52.4 (17.7–172.2)	24.6 (10.7–58.0)	67.3 (28.8–213.5)	<0.0001
Procalcitonin, μg/L	7.7 (1.4–28.8)	1.5 (0.3–7.2)	13.6 (2.9–37.8)	<0.0001
C-reactive protein, mg/L	222 (141–298)	201 (134–275)	222 (152–302)	0.295
Biochemistry				
Leukocyte count, 10^9^/L, highest value	16.9 (10.4–23.9)	15.5 (9.7–22.7)	17.6 (12.2–24.5)	0.197
Na^+^, mmol/L, lowest value	136 (132–138)	136 (134–139)	135 (131–138)	0.198
K^+^, mmol/L, highest value	4.3 (4.0–4.9)	4.1 (3.8–4.3)	4.5 (4.1–5.2)	<0.0001
Glucose, mmol/L, highest value	8.3 (7.1–11.5)	7.5 (5.8–9.9)	8.6 (7.3–12.4)	0.011
Creatinine, μmol/L, highest value	119 (77–205)	82 (58–127)	142 (82–229)	0.002
Hemoglobin, mmol/L, lowest value	5.7 (4.9–6.5)	5.7 (4.9–6.7)	5.7 (4.8.9–6.3)	0.429
pH, lowest value	7.30 (7.20–7.37)	7.35 (7.29–7.40)	7.28 (7.18–7.34)	0.006
pO_2_, kPa, from lowest PaO_2_/FiO_2_ ratio	13.7 (10.9–19.7)	14.4 (11,5–22.3)	12.8 (10.8–18.1)	0.137
Base excess, mmol/L, lowest value	−5.5 (−9.4 to −1.8)	−2.2 (−5.7 to −2.2)	−6.7 (−10.9 to −2.5)	0.001
Lactate, mmol/L, highest value	1.9 (1.1–4.1)	1.0 (0.7–1.6)	2.5 (1.5–5.0)	<0.0001
ICU scoring systems and treatment				
SAPS II^a^	45 (35–52)	35 (29–48)	46 (39–56)	0.001
SOFA (day 1)^b^	7 (4–10)	3 (2–5)	8 (7–10)	<0.0001
LRINEC^c^	8 (6–9)	8 (5–9)	8 (6–10)	0.103
ICU admission^d^	123 (91)	28 (72)	95 (99)	<0.0001
Ventilator treatment	122 (90)	27 (70)	95 (99)	<0.0001
Renal replacement therapy^e^	34 (25)	5 (13)	29 (30)	0.035
Amputation of limb or penis^e^	27 (20)	5 (13)	22 (23)	0.184
Mortality				
28-day (%, 95 % CI)	22 (16, 11–24)	8 (21, 11–36)	14 (15, 9–23)	0.398
90-day (%, 95 % CI)	31 (23, 17–31)	9 (23, 12–39)	22 (23, 16–32)	0.984
180-day (%, 95 % CI)	36 (27, 20–35)	10 (26, 14–41)	26 (27, 19–37)	0.864
Long-term up to 2.5 years^f^ (%, 95 % CI)	44 (33, 25–41)	12 (31, 18–47)	32 (33, 25–43)	0.773

Values denote median (interquartile range) or number (%). Differences between the shock and non-shock group were tested using Mann-Whitney *U* or χ^2^-test/Fisher’s exact test

^a^Number of patients with missing values: n = 5 (n = 3 for nonshock; n = 2 for shock)

^b^Number of patients with missing values: n = 6 (n = 3 for nonshock; n = 3 for shock)

^c^Number of patients with missing values: n = 15 (n = 9 for nonshock; n = 6 for shock)

^d^Three patients died before admission to the ICU (n = 2 for nonshock; n = 1 for shock)

^e^Within the first 7 days in the ICU

^f^A follow-up up to 2.5 years

*CI* Confidence interval, *ICU* Intensive care unit, *LRINEC* Laboratory risk indicator for necrotizing fasciitis, *SAPS* Simplified Acute Physiology Score, *SOFA* Sequential Organ Failure Assessment

### Association between pentraxin-3 and disease severity

**Fig. 2:**
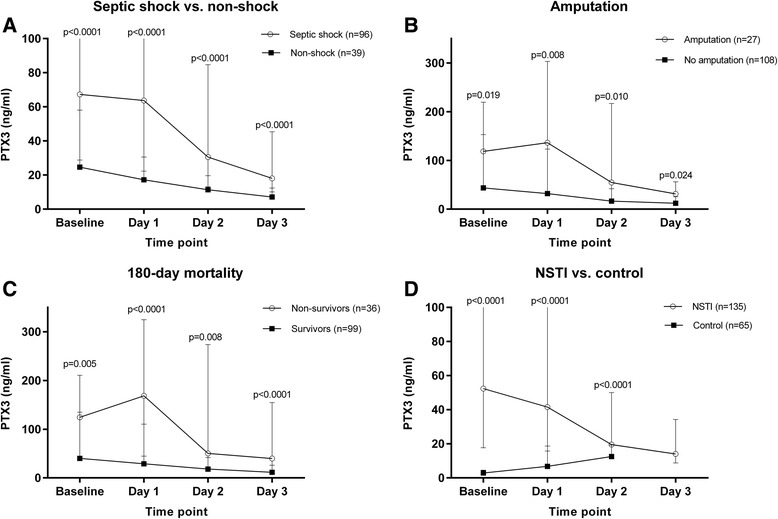
Pentraxin-3 level upon admission (baseline) and for the following 3 days in **a** septic shock versus nonshock, **b** amputation versus no amputation, **c** 180-day mortality and **d** NSTI versus control. *NSTI* Necrotizing soft tissue infection, *PTX3* Pentraxin-3

Baseline PTX3 level was significantly higher in patients with septic shock (67.3 (IQR, 28.8–213.5) versus 24.6 (IQR, 10.7–58.0) ng/mL, *p* < 0.0001) (Fig. 2a). Moreover, baseline PTX3 level was significantly higher in patients who underwent amputation during the first 7 days in the ICU (118.6 (IQR, 42.4–219.6) versus 43.6 (IQR, 15.5–153.1) ng/mL, *p* = 0.019) (Fig. 2b) and significantly higher in patients who died within the first 180 days after admission (124.3 (IQR, 43.1–210.8) versus 40.0 (IQR, 14.6–134.9) ng/mL, *p* = 0.005) (Fig. 2c).

In general, the PTX3 level decreased during admission to the ICU, but it increased over the first day for those with a fatal outcome whilst the PTX3 levels decreased in survivors (168.3 (IQR, 44.9–324.7) versus 29.0 (IQR, 14.2–110.4) ng/mL, *p* < 0.0001). A similar pattern was observed in patients who underwent amputation versus no amputation (136.3 (IQR, 32.6–303.3) versus 32.0 (IQR, 14.7–123.2) ng/mL, *p* = 0.008). Patients with a streptococcal infection had a higher baseline PTX3 level (93.9 (IQR, 22.1–283.5) versus 44.7 (IQR, 16.5–130.3) ng/mL, *p* = 0.035). No difference was observed between mono- and polymicrobial infection (44.0 (IQR, 16.9–169.5) versus 65.2 (IQR, 19.8–174.3) ng/mL, *p* = 0.346). Lastly, the patients with NSTI had a significantly higher baseline PTX3 level compared with control patients without infection (52.4 (IQR, 17.7–172.2) versus 2.9 (IQR, 2.0–4.5) ng/mL, *p* < 0.0001) (Fig. 2d).

### Procalcitonin and C-reactive protein

Baseline PCT level was significantly higher in patients with septic shock (13.6 (IQR, 2.9–37.8) versus 1.5 (IQR, 0.3–7.2) μg/L, *p* < 0.0001), whilst no significant difference in baseline CRP level was observed (222 (IQR, 152–302) versus 201 (IQR, 134–275) mg/mL, *p* = 0.295) (Table 2). There were no significant differences in baseline PCT or CRP levels according to amputation (25.2 (IQR, 1.9–78.5) versus 7.0 (IQR, 1.4–22.7) μg/L, *p* = 0.060 and 202 (IQR, 90–244) versus 225 (IQR, 143–307) mg/L, *p* = 0.123), respectively.

PTX3 correlated with PCT (Rho = 0.64 (95 % CI, 0.52–0.73), *p* < 0.0001) and SAPS II (Rho = 0.45 (95 % CI, 0.30–0.58), *p* < 0.0001) but not with CRP (Rho = 0.15 (95 % CI, −0.03–0.32), *p* = 0.095). Moreover, baseline PTX3 correlated with the Sequential Organ Failure Assessment (SOFA) score (Rho = 0.48 (95 % CI, 0.34–0.60), p < 0.0001) and baseline lactate level (Rho = 0.57 (95 % CI, 0.44–0.67), *p* < 0.0001). PTX3 also correlated with creatinine (Rho = 0.46 (95 % CI, 0.31–0.58), *p* < 0.0001) and patients receiving renal replacement therapy within the first 7 days in the ICU had a higher baseline PTX3 level (141.2 (IQR, 55.0–276.4) versus 34.5 (IQR, 13.0–124.3) ng/mL, *p* < 0.0001).

### Univariate survival analysis

**Fig. 3:**
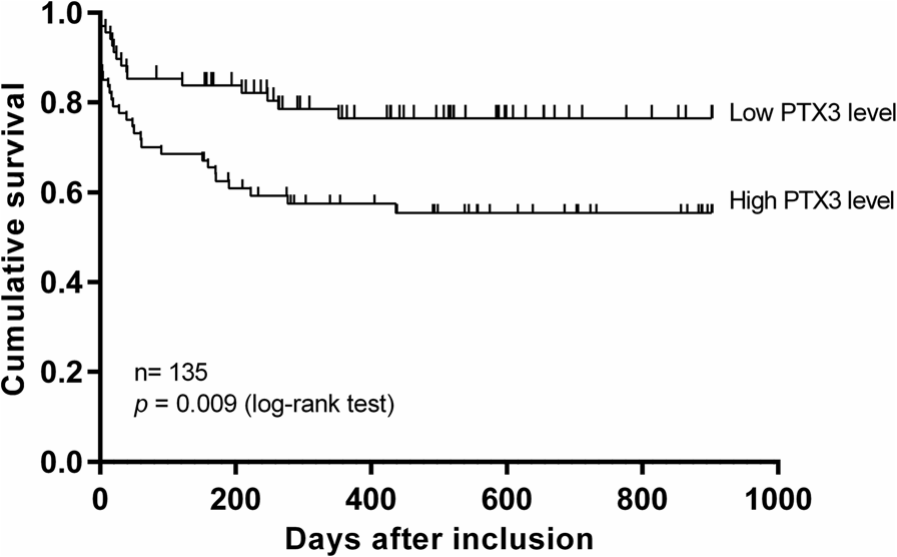
Kaplan-Meier curves of long-term mortality up to 2.5 years in patients with necrotizing soft tissue infections stratified by median plasma PTX3 level (>52.4 ng/mL). *PTX3* Pentraxin-3

During a median follow-up of 17 months (range 3–30 months), 44 patients died (32 with septic shock upon admission). Baseline PTX3 level above the median of 52.4 ng/mL was associated with lower survival rate (*p* = 0.009) (Fig. 3). Univariate survival analysis revealed that age, SAPS II, PTX3, PCT and CRP were significantly associated with 180-day mortality (Table 3).

**Table 3:** Cox univariate analysis for 180-day mortality

Variable	n	180-day mortality		
		HR	95 % CI	*p*
Age, years	135	1.04	1.01–1.07	0.005
SAPS II, point	130	1.06	1.05–1.08	<0.0001
Sex				
Female	51	ref.		
Male	84	1.42	0.70–2.89	0.331
Chronic disease				
No	49	ref.		
Yes	86	1.81	0.85–3.84	0.124
Pentraxin-3				
Low ≤52.4 ng/mL		ref.		
High >52.4 ng/mL		2.60	1.28–5.29	0.008
Procalcitonin				
Low ≤7.73 μg/L		ref.		
High >7.73 μg/L		2.22	1.11–4.44	0.024
C-reactive protein				
Low ≤222 mg/L		ref.		
High >222 mg/L		0.44	0.22–0.89	0.021

Markers are divided by high versus low concentrations according to median values

*CI* Confidence interval, *HR* Hazard ratio, *ref.* Referent, *SAPS II* Simplified Acute Physiology Score II

### Multivariate survival analysis

When adjusted for variables specified in our protocol [28], the association between high baseline PTX3 level and 180-day mortality was not significant (hazard ratio (HR) 1.05 (95 % CI, 0.47–2.35), *p* = 0.902) (Table 4). The same lack of association was observed for PCT (HR 0.86 (95 % CI, 0.38–1.95), *p* = 0.713) and CRP (HR 0.71 (95 % CI, 0.34–1.51), *p* = 0.376).

**Table 4:** Multivariate analysis by Cox proportional hazards regression model and diagnostic accuracy of 180-day mortality

Markers	180-day mortality									
	HR^a^	95 % CI	*p*	Sensitivity	95 % CI	Specificity	95 % CI	ROC-AUC	95 % CI	*p*
PTX3	1.05	0.47–2.35	0.902	0.69	0.53–0.82	0.56	0.50–0.60	0.66	0.56–0.76	0.005
PTX3^b^	1.34	0.60–3.00	0.480	0.67	0.51–0.80	0.68	0.62–0.72	.	.	.
PCT	0.86	0.38–1.95	0.713	0.66	0.50–0.79	0.55	0.49–0.60	0.65	0.55–0.76	0.007
CRP	0.71	0.34–1.51	0.376	0.34	0.21–0.50	0.41	0.36–0.47	0.32	0.21–0.43	0.001
PTX3 + PCT	.	.	.	.	.	.	.	0.66	0.56–0.76	0.005
PTX3 + CRP	.	.	.	.	.	.	.	0.72	0.61–0.82	<0.0001
PCT + CRP	.	.	.	.	.	.	.	0.71	0.60–0.82	<0.0001
PTX3 + PCT + CRP	.	.	.	.	.	.	.	0.72	0.62–0.83	<0.0001

Hazard ratio, sensitivity and specificity are calculated for high (above median) versus low (below median) baseline levels of the inflammatory biomarkers. Five patients are not included in the analysis due to missing data regarding Simplified Acute Physiology Score II

^a^Adjusted for age, sex, Simplified Acute Physiology Score II and chronic disease (yes/no)

^b^PTX3 dichotomized by the optimal cutoff (69.8 ng/ml) found by the ROC curve (highest sum of sensitivity and specificity)

*CRP* C-reactive protein, *HR* Hazard ratio, *PCT* Procalcitonin, *PTX3* Pentraxin-3, *ROC-AUC* Receiver operating characteristic-area under the curve, *CI* Confidence interval

Dots: Values cannot be given

### Diagnostic accuracy of the inflammatory biomarkers to predict 180-day mortality

We found low levels of sensitivity and specificity for PTX3, PCT and CRP in the analysis of 180-day mortality when the medians were used to define high versus low baseline levels in plasma (Table 4).

**Fig. 4:**
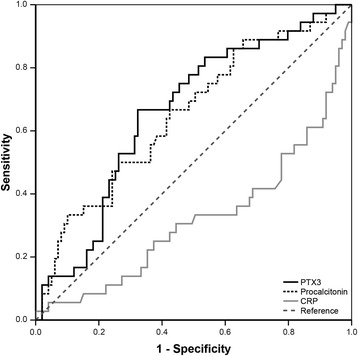
Receiver operating characteristic curve of 180-day mortality in patients with necrotizing soft tissue infections for the inflammatory biomarkers. *CRP* C-reactive protein, *PTX3* Pentraxin-3

The AUC for the ROC was low for PTX3 levels (AUC = 0.66, *p* = 0.005), as was the case for PCT levels (AUC = 0.65, *p* = 0.007) and CRP levels (AUC = 0.32, *p* = 0.001) (Fig. 4). The AUC improved when the inflammatory markers were combined (AUC = 0.72, *p* < 0.0001). The optimal cutoff for PTX3 was 69.8 ng/mL, identified as the value corresponding to the maximum sum of sensitivity (0.67) and specificity (0.68). When the multivariate analysis was performed with the optimal cutoff instead of the median, the risk of 180-day mortality in patients with a high baseline PTX3 level increased but was still not significant (HR 1.34 (95 % CI, 0.60–3.00), *p* = 0.480) (Table 4).

## Discussion

We found that a high PTX3 level in patients with NSTI at time of admission was associated with septic shock, renal replacement therapy, amputation and risk of death. High PCT and CRP levels were not associated with amputation. Moreover, baseline PTX3 level correlated with SAPS II, SOFA score, lactate and creatinine. None of the inflammatory biomarkers was significantly associated with 180-day mortality in the multivariate analysis and there was a low sensitivity, specificity and AUC. However, the AUC improved when the markers were combined.

PTX3 levels have been shown to be 1–2 ng/mL in healthy individuals [32, 33]. In a recent study using the same assay, healthy individuals had a median PTX3 level of 3.5 ng/mL (range, 0.0–8.3 ng/mL) [29] compared with 2.9 ng/mL (range, 1.7–28.5 ng/mL) in the control patients in this study. The wider range might be explained by our control patients being matched by age and sex, thereby giving older controls with comorbidities that contribute to increased PTX3 levels. Nevertheless, we found significantly higher levels in the patients with NSTI underlining PTX3 as a marker of bacterial infection. However, PTX3 is not a specific marker for bacterial infections, which is why we adjusted our analysis for comorbidities, such as cardiovascular disease and malignancy, where high PTX3 levels have been shown to be correlated with disease severity and mortality [14, 15, 18, 34]. When adjusted for age, sex, chronic disease and SAPS II, the association between high PTX3 level upon admission and 180-day mortality was not significant, indicating that PTX3 is not an independent predictor of 180-day mortality. The same was observed for PCT and CRP. In line with this, we did not find a difference in mortality between septic shock versus nonshock patients (Table 2), which could otherwise be expected. This probably reflects that we did not include enough patients to demonstrate a difference in an outcome with low incidence.

It should be noted that the ROC-AUC for 180-day mortality was low but comparable to a previous study investigating PTX3, PCT and CRP in emergency room patients with suspected infection [35]. It confirms that these inflammatory biomarkers should be incorporated as part of an overall assessment of patients with NSTI rather than instead of clinical assessment. This is in line with previous studies investigating patients with sepsis or septic shock that found PCT and CRP to have limited abilities to predict outcome [36–38]. However, we found that the AUC improved when the inflammatory markers were combined. It may indicate that PTX3 in combination with other inflammatory markers can be used to discern severe NSTI from milder courses in order to refine the risk stratification. In particular, PTX3 level was higher in patients needing amputation during the first 7 days in the ICU, whereas PCT and CRP were not. Therefore, PTX3 might be used to identify the high-risk patients who require aggressive surgery, while directing a conservative approach to low-risk patients. The correlation between PTX3 and SAPS II may also suggest that PTX3 can be used as an easy assessment of disease severity until SAPS II can be calculated or in cases where SAPS II cannot be calculated due to missing values. An explanation for the association between high PTX3 level and increased disease severity and mortality may be found in the biological action of PTX3 as it is released in response to proinflammatory stimuli from interleukin-1 and tumor necrosis factor, thus potentially reflecting higher bacterial loads. PTX3 also acts as a modulator of the complement system, but a pathophysiological role related to tissue damage by amplifying the complement pathways remains to be elucidated.

To our knowledge, this is one of the largest prospective observational study cohorts of patients with NSTI and the first study to investigate the prognostic value of PTX3 in patients with NSTI. It is a strength that we managed to include 98 % of all patients transferred to our center where the treatment of NSTI has been centralized at a national level and that multiple measurements from each patients were obtained. Moreover, this study was based on standardized sampling procedures ensured by using only a few individuals as part of a 24-h on-call team with nine members. Lastly, the external validity of the study results is high due to few inclusion and exclusion criteria, thus increasing the chance of the study cohort representing the vast majority of patients with NSTI and making the results applicable to daily clinical settings.

Several limitations must be considered. Firstly, the study might be subject to potential bias as a consequence of the inability to control for unknown confounders. Patients with NSTI represent a complex study population as seen by the range and variance in age, microbiologic agents, site of infection and comorbidities, thus making it difficult to isolate factors that potentially affect the immunological response. Importantly, these factors were not significantly different between patients with septic shock and the other patients. Secondly, we might have been able to minimize risk of observer and detection bias if the individuals who reviewed the medical records and did the statistical analyses had been blinded. However, the staff performing the laboratory analyses of PTX3, PCT and CRP were blinded to study purpose and patient outcome. Thirdly, almost one out of 10 patients did not have sepsis according to the international criteria by Bone et al. [30] (Fig. 1). This relatively large proportion might be explained by the fact that most patients had to be transferred to our center to be included. Some patients might have died before transfer or might have been too hemodynamically unstable to be transferred, thereby increasing the risk of selection bias. However, infections in critically ill patients can also be present without any clinical manifestation of the systemic inflammatory response syndrome [39], which is confirmed in this study cohort. It is crucial that clinicians know that patients with NSTI might not show the classical signs of sepsis. Lastly, it would have been interesting to have local PTX3 measurements from the focus of infection and compare this with the released amount in the blood stream. This could be incorporated in future study protocols as it, to our knowledge, has not been investigated before.

This study indicates that clinicians may consider PTX3 as a marker of infection on equal terms with PCT and that these markers are more reliable as prognostic markers of disease severity than is CRP in patients with NSTI. Additionally, these findings might be useful in creating homogenous patient groups for future clinical trials investigating NSTIs. It is important to note that multiple analyses were conducted and that PCT and CRP were secondary outcomes. This increases the risk of chance findings. Even though we found associations between PTX3 and clinical outcomes, the results should be interpreted with caution. The prognostic and predictive values need to be assessed in larger studies before PTX3 can be implemented in daily clinical practice. However, the results imply a potential role for PTX3 in severity and mortality prognostication in patients with NSTI.

## Conclusions

We found that plasma PTX3 levels were associated with septic shock, renal replacement therapy, amputation and risk of death in patients with NSTI. However, PTX3 was not an independent predictor of 180-day mortality.

## Key messages

- The largest to date prospective, observational study of patients with NSTI sampled over 25 months with up to 2.5-year follow-up.
- It is of topical interest that we now have a new inflammatory biomarker with a potential future role in severity and mortality prognostication in patients with NSTI.
- PTX3 level was higher in patients with NSTI needing amputation, whereas PCT and CRP were not. Therefore, PTX3 might be used to identify the high-risk patients who require aggressive surgery, while directing a conservative approach to low-risk patients.
- PTX3 is more reliable as a prognostic marker of disease severity than PCT and CRP in patients with NSTI.
- It is crucial that clinicians know that one out of 10 patients with NSTI might not show the classical signs of sepsis.

## Abbreviations

AUC: Area under the curve
CI: Confidence interval
CRP: C-reactive protein
ELISA: Enzyme-linked immunosorbent assay
HZ: Hazard ratio
ICU: Intensive care unit
IQR: Interquartile range
NSTI: Necrotizing soft tissue infection
PCT: Procalcitonin
PTX3: Pentraxin-3
ROC: Receiver operating characteristic
SAPS II: Simplified Acute Physiology Score II
SOFA: Sequential Organ Failure Assessment
